# Immunological Avalanches in Renal Immune Diseases

**DOI:** 10.3390/biomedicines13041003

**Published:** 2025-04-21

**Authors:** Davide Viggiano, Pietro Iulianiello, Antonio Mancini, Candida Iacuzzo, Luca Apicella, Renata Angela Di Pietro, Sarah Hamzeh, Giovanna Cacciola, Eugenio Lippiello, Andrea Gigliotti, Carmine Secondulfo, Giancarlo Bilancio, Giuseppe Gigliotti

**Affiliations:** 1 Department Translational Medical Sciences, University of Campania, 80131 Naples, Italy; iulianiellopietro@live.com (P.I.); giovanna.cacciola.gc@gmail.com (G.C.); 2 Department of Nephrology and Dialysis, Eboli Hospital, 84025 Eboli, Italy; antonellomancini130986@gmail.com (A.M.); andreagigliotti73@yahoo.it (A.G.); g.gigliotti@aslsalerno.it (G.G.); 3 Unit of Nephrology, Dialysis and Transplantation, Salerno University Hospital, 84131 Salerno, Italy; candida.iacuzzo@sangiovannieruggi.it (C.I.); lucaapicella@gmail.com (L.A.); angela.dipietro@sangiovannieruggi.it (R.A.D.P.); 4 Department of Public Health, Federico II University of Naples, 80131 Naples, Italy; sarahhamzeh1@gmail.com; 5 Department Mathematics and Physics, University of Campania, 81100 Caserta, Italy; eugenio.lippiello@unicampania.it; 6 Department of Medicine, Surgery and Dentistry “Scuola Medica Salernitana”, University of Salerno, 84081 Baronissi, Italy; csecondulfo@unisa.it (C.S.);

**Keywords:** acute kidney injury, lupus, proteinuria, critical state

## Abstract

The complex nature of immune system behavior in both autoimmune diseases and transplant rejection can be understood through the lens of avalanche dynamics in critical-point systems. This paper introduces the concept of the “immunological avalanche” as a framework for understanding unpredictable patterns of immune activity in both contexts. Just as avalanches represent sudden releases of accumulated potential energy, immune responses exhibit periods of apparent stability followed by explosive flares triggered by seemingly minor stimuli. The model presented here draws parallels between immune system behavior and other complex systems such as earthquakes, forest fires, and neuronal activity, where localized events can propagate into large-scale disruptions. In autoimmune conditions like systemic lupus erythematosus (SLE), which affects multiple organ systems including the kidneys in approximately 50% of patients, these dynamics manifest as alternating periods of remission and flares. Similarly, in transplant recipients, the immune system exhibits metastable behavior under constant allograft stimulation. This critical-point dynamics framework is characterized by threshold-dependent activation, positive feedback loops, and dynamic non-linearity. In autoimmune diseases, triggers such as UV light exposure, infections, or stress can initiate cascading immune responses. In transplant patients, longitudinal analysis reveals how monitoring oscillatory patterns in blood parameters and biological age markers can predict rejection risk. In a preliminary study on kidney transplant, all measured variables showed temporal instability. Proteinuria exhibited precise log–log linearity in power law analysis, confirming near-critical-point system behavior. Two distinct dynamic patterns emerged: large oscillations in eGFR, proteinuria, or biological age predicted declining function, while small oscillations indicated stability. During avalanche events, biological age increased dramatically, with partial reversal leaving persistent elevation after acute episodes. Understanding these dynamics has important implications for therapeutic approaches in both contexts. Key findings suggest that monitoring parameter oscillations, rather than absolute values, better indicates system instability and potential avalanche events. Additionally, biological age calculations provide valuable prognostic information, while proteinuria measurements offer efficient sampling for system dynamics assessment. This conceptual model provides a unifying framework for understanding the pathogenesis of both autoimmune and transplant-related immune responses, potentially leading to new perspectives in disease management and rejection prediction.

## 1. Introduction

Most current clinical approaches to pathologies rely on discontinuous measurements that provide only static snapshots. Clinicians and scientists typically extract information about change by comparing variables between separate measurements. Only rarely do we capture high-frequency measurements over extended periods, as with Holter monitors for blood pressure, ECG, EEG, or more recently, continuous glucose monitoring.

However, many pathological states are inherently dynamic conditions with complex temporal patterns. Therefore, a single measurement—akin to one photograph of clouds to predict rain—is fundamentally inadequate. Just as weather forecasting requires temporal data, measuring cardiac electrical potential at a single instant rather than evaluating its pattern over time provides limited insight.

Some pathologies exhibit complex and sometimes unpredictable internal dynamics, even with appropriate therapy and lifestyle modifications. Conditions involving the immune system, such as lupus or transplant recipients, exemplify this challenge. In these conditions, the static snapshots of a few immune parameters cannot help clinicians predict imminent instability, such as a lupus flare or acute transplant rejection. In physics, the study of phenomena where small stimuli trigger large-scale cascading events—like landslides, electrical grid failures, or epileptic seizures—is addressed by statistical physics and complexity theory (e.g., maximum Lyapunov exponent [1]). Among these conceptual frameworks, avalanche theory offers valuable insights. A simple model illustrating this mathematical theory is a sand pile: imagine randomly adding sand grains to a surface. When the pile exceeds a critical threshold, it collapses, spreading grains to adjacent areas—creating an avalanche. The crucial insight is that near this critical point, adding just one more grain can dramatically transform the seemingly stable mound. This represents chaotic behavior, or the system’s extreme sensitivity to initial conditions.

The complex behavior of the immune system in kidney transplant patients is analogous to avalanche dynamics in critical-point systems, characterized by periods of stability punctuated by sudden immune activity flares.

This study introduces the concept of the “immunological avalanche” as a framework for understanding the unpredictable patterns of transplant rejection. Like complex systems such as earthquakes and neural networks, transplanted patients’ immune systems comprise a myriad of interacting immune cells under constant allograft stimulation [2]. These systems exhibit metastable behavior: long periods of apparent equilibrium punctuated by sudden, large-scale immune responses triggered by minimal perturbations. This critical-point dynamic helps explain the unpredictable patterns of transplant rejection, where minor immunological triggers can cascade into systemic responses. This study investigates how longitudinal analysis of blood parameters can reveal critical-point indicators for predicting transplant rejection risk in kidney recipients. We examine the oscillatory patterns of multiple blood markers while simultaneously analyzing an estimate of DNA methylation patterns in peripheral cells to assess both chronological and biological age. By identifying “avalanche-like” dynamics in these parameters, we aim to develop an early warning system for predicting future rejection events. This approach combines traditional biomarker monitoring with epigenetic clock analysis to create a more comprehensive risk assessment model.

## 2. Oscillating Diseases

Many chronic diseases exhibit oscillatory behavior characterized by alternating periods of intense activity (flares) and relative quiescence (remission). This dynamic pattern emerges across multiple biological systems through similar underlying mechanisms of interacting networks reaching critical states. In immune system disorders, conditions such as rheumatoid arthritis, systemic lupus erythematosus [3], and multiple sclerosis [4] demonstrate periodic flares driven by complex interactions between immune cells, cytokines, and tissue responses [5,6,7]. Within the nervous system, epilepsy and migraine show rhythmic patterns of hyperactivity and recovery, mediated by neural circuit excitability and neurotransmitter dynamics [8]. Hematological disorders involving erythrocytes, such as sickle cell disease, display cyclical crises influenced by cell rheology and vascular interactions [9,10]. The coagulation cascade and platelet system exhibit oscillatory behavior in conditions like thrombotic thrombocytopenic purpura and immune thrombocytopenia, where platelet counts fluctuate through autoimmune mechanisms [11]. Somehow, deep vein thrombosis and myocardial ischemia may also represent special forms of oscillating diseases involving the coagulation cascade. Complement system disorders, including paroxysmal nocturnal hemoglobinuria, show periodic exacerbations linked to complement activation cycles [12]. These pathologies share a common thread: their oscillations emerge from the complex interactions of system components that reach critical states, often following power law distributions in their temporal patterns. Understanding these oscillatory dynamics is crucial for developing targeted therapeutic interventions that could potentially dampen or prevent disease flares. Recent advances in network analysis and systems biology have revealed that these seemingly distinct conditions may share fundamental principles in how their underlying biological networks generate periodic behavior, suggesting potential common therapeutic approaches across different organ systems (see Table 1 for a list of oscillating diseases).

## 3. The Framework of Avalanche

The oscillatory nature of many chronic diseases suggests an underlying organizational principle that can be understood through the framework of critical-state networks and biological avalanches. These systems operate at a critical point between order and chaos, emerging from a delicate balance between activating and inhibitory signals. In this state, small perturbations can trigger responses across multiple scales.

Four essential elements characterize these systems:

1. A complex system composed of several interacting parts.
2. Non-linear interactions among elements.
3. A weak driving mechanism (bias force) applied to select units, which gradually increases the system’s potential energy until reaching a threshold that triggers an avalanche.
4. An inhibitory system that stops the avalanche.

This critical-state behavior manifests through several key characteristics:

  (a) Power Law Scaling: The frequency of immune events decreases as a power of the event size, meaning small immune responses are common while large-scale responses (avalanches) are rare but significant. Unlike systems with “characteristic” sizes, immune responses can range from localized cellular activity to massive system-wide engagement, allowing for proportional responses to threats.
  (b) Scale-Free Properties: The network exhibits similar statistical patterns at different scales, with most nodes (cells) having few connections while certain hubs (like regulatory T cells or memory cells) are highly connected. This structure makes the system robust against random perturbations but vulnerable to disruptions of hub cells, potentially explaining the emergence of certain immune disorders.
  (c) Metastability: The system alternates between periods of relative calm (remission) and abrupt activity bursts (flares), a pattern characteristic of many chronic immune-mediated diseases.

The mathematical framework of avalanches differs from existing models of immune threshold activation. Indeed, in avalanche theory, the existence of a threshold mechanism is just one of the key ingredients that typically govern avalanche-like dynamics. Two additional elements are essential: the presence of many interacting components and a slow driving mechanism. Furthermore, to obtain a true threshold mechanism controlling the transition from a stable to an unstable state (avalanche), the interactions among elements must be non-linear.

As will be discussed below, the immune system is a candidate for such a structure, the hubs being key immune cells like regulatory T cells or memory B cells that coordinate responses.

## 4. Avalanches in Immune Disease: Overview

Several diseases exhibit behaviors consistent with the immunological avalanche model, where immune activity alternates between calm periods and flares.

As reported in Table 2, various immunological diseases have triggers that at the end, by increasing the potential energy in the immune system close to the threshold of the overall activation, lead to recurrent episodes of “flares”. For example, SLE is triggered by UV light or infections or other stressors [54,55], with the formation of autoantibodies and a cascade activation of complements [56,57,58,59]. This is similar to several other diseases with a natural history of flares, such as multiple sclerosis, asthma, and inflammatory bowel diseases.

This cyclical behavior closely resembles the avalanche phenomenon, where systems oscillate between stability and instability based on inputs.

Indeed, the immune system exemplifies this framework through a three-stage process: (i) Trigger Event: Specific stimuli (infections, microorganisms, or dysbiosis) can initiate an autoimmune response. Also, dysbiosis can have a role in autoimmune diseases [63]. (ii) Cascade Propagation: Immune cells activate beyond a threshold, recruiting additional immune components [64,65]. (iii) Systemic Impact: The response escalates into widespread inflammation and tissue damage.

The scale-free nature would make the immune system robust to random perturbations but vulnerable to attacks on hubs. This could explain why certain immune disorders arise when highly connected cells are compromised.

This framework offers several clinical insights:

- Disease management might be more effective by keeping the system slightly away from its critical point rather than attempting complete suppression.
- Network modulation could prove more beneficial than targeting individual molecular pathways.
- Patient response variability to treatments might be explained by their network’s proximity to criticality.
- Early warning signals, similar to seismological precursors, might help predict major flares.

Understanding diseases through critical-state networks provides a mathematical foundation for predicting intervention outcomes while explaining both the unpredictability of individual flares and the predictable statistical patterns in their distribution.

To address the complex dynamics of immune system instability in recurrent autoimmune diseases, we propose a framework based on triggers, positive feedback loops, and inhibitors, drawing parallels with neuronal avalanches.

In Table 3, we summarize potential triggers and inhibitors of the immune system.

The immune system characterization may employ a multi-modal strategy encompassing both traditional and novel biomarkers. At the molecular level, cytokine profiles focusing on the balance between pro-inflammatory (IL-6, TNF-α, IL-1β) and anti-inflammatory mediators, alongside classic inflammation markers such as CRP and ESR, can be used.

The immune activation status might be assessed through autoantibody levels and lymphocyte subset analysis, while regulatory capacity is evaluated via Treg functionality and regulatory cytokine levels (IL-10, TGF-β). This core immunological assessment is complemented by analysis of oxidative stress markers and gut microbiota profiling, recognizing the critical role of host–microbiome interactions in immune regulation.

The predictive component of this framework employs dynamic systems analysis to identify early warning signals of impending flares. Network-based modeling represents immune interactions as interconnected nodes, enabling simulation of perturbation propagation through the system. This approach is enhanced by machine learning algorithms trained on longitudinal biomarker data, creating predictive models for disease trajectories. The framework includes the development of dynamic thresholds for key biomarker ratios, offering quantitative measures of system stability.

Stabilization strategies are structured in a hierarchical intervention model. Primary interventions focus on trigger reduction through infection control, dietary modification, and microbiota restoration. Secondary approaches enhance regulatory mechanisms through pharmacological interventions, lifestyle modifications, and experimental immunotherapies. The framework emphasizes continuous monitoring through emerging technologies, enabling real-time tracking of inflammatory markers and rapid intervention adjustment.

This integrated approach represents a shift from traditional linear thinking about immune dysfunction toward a network-based understanding of disease dynamics. By considering the immune system as a critical-state network, we can better understand how local perturbations may propagate to cause systemic effects, and how strategic interventions might prevent or minimize disease flares. Initial clinical applications of this framework have demonstrated improved prediction of disease exacerbations and more effective intervention timing, though larger-scale validation studies are ongoing. Future developments will focus on incorporating additional biomarkers and refining predictive algorithms through machine learning approaches trained on expanded longitudinal datasets.

## 5. Immunological Avalanche in Lupus Nephritis

Systemic lupus erythematosus (SLE) is a complex and challenging autoimmune disease that can affect almost any organ in the body. For unclear reasons, it has a wide range of clinical presentations such as joint pain or a skin rash, and complications involving the kidneys, blood, or nervous system.

Kidney involvement is clinically apparent in approximately 50% of SLE patients and is a significant cause of morbidity and mortality [82]. The clinical presentation of lupus nephritis is highly variable, ranging from asymptomatic hematuria and/or proteinuria to nephrotic syndrome and rapidly progressive glomerulonephritis with loss of kidney function [83].

Just as in avalanches there is a state of calm where a trigger releases the potential energy stored in the snow, so too in autoimmune diseases. A seemingly minor stimulus can unleash a cascade of immune responses, transforming a stable phase into an explosive flare-up of inflammation and tissue damage.

This process shares similarities with other complex systems where simple units with thresholds accumulate energy and then release it back into the system. Examples of such systems include earthquakes, forest fires [84], and nuclear chain reactions but also neuronal activity [8]. In these cases, when one unit surpasses its threshold, it triggers a chain reaction, causing other units to activate in succession and initiating a cascade that spreads throughout the entire system.

### 5.1. Definition and Epidemiology of Systemic Lupus Erythematosus (SLE)

The term “Lupus” was first used in the Middle Ages by the surgeon Rogerius Salernitanus (1140–c. 1195), who practiced medicine at the Salerno Medical School, near Eboli, to describe disfiguring skin lesions on the face and ulcers on the limbs, similar to “wolf bites” [85]. Drawing on this terminology, the French dermatologist Biett, in the early 19th century, coined the term “Lupus Erythematosus” to describe the erythematous, hard, and scaly skin lesions that disfigured the face of one of his patients [86]. Later, the American physician William Osler was the first to recognize that it was a systemic disease. In the late 19th century, he coined the term “Systemic Lupus Erythematosus” (SLE) [86]. A major turning point in the history of SLE occurred in 1948, when Dr. Malcolm M. Hargraves and Mayo Clinic associates Helen Richmond and Dr. Robert Morton Mayo described L.E. cells, the first diagnostic test that paved the way for the search for many other diagnostic markers, including antibodies in the serum of SLE patients, especially the anti-DNA antibody [87].

**Figure 1:**
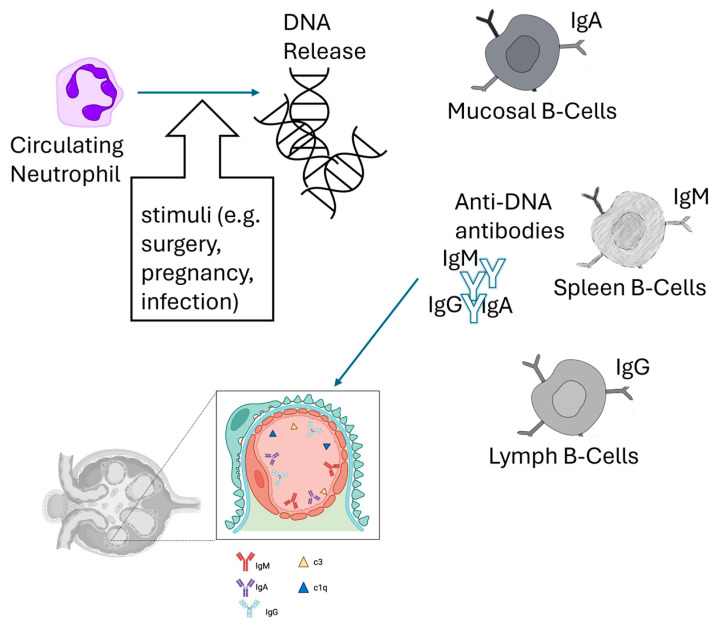
A schematic diagram of the pathogenesis of lupus nephritis. Subtle stimuli activate DNA release from circulating cells such as neutrophils (NETosis), which prompts the formation of immunocomplexes with different immunoglobulin isotypes. The immunocomplexes reach the kidney glomeruli, thereby activating the complement system (C3 and C1q) and causing glomerular damage.

Today, systemic lupus erythematosus (SLE) is known as a chronic systemic autoimmune disease of unknown etiology, caused by the loss of immunological tolerance confirmed by the presence of autoantibodies against endogenous nuclear cellular material [88]. This condition determines a multi-system immune response [88], a real “immunological avalanche” that causes damage to different organs and tissues (see Figure 1).

The prevalence of SLE varies between 4 and 50 cases per 100,000 inhabitants. It is more common in females, with a ratio of approximately 1:9 compared to males, and the peak onset usually occurs between the ages of 30 and 40, although cases of early or late onset are possible [89]. African-American and Asian ethnic groups are more frequently affected by aggressive and early-onset clinical pictures compared to Caucasians. Predisposing factors include female gender, hormones (especially estrogen), heredity, and many environmental triggers including ultraviolet light (the most recognized), drugs/supplements (echinacea, trimethoprim/sulfamethoxazole), smoking, infections (especially Epstein–Barr virus), silica, mercury, and others. Psychological stress has also been linked to a 50% increased risk of developing lupus [90].

SLE can potentially affect any organ system, but kidney involvement, called lupus nephritis, is particularly common. Approximately 25% to 50% of patients with SLE have kidney involvement at onset, and up to 60% of adults develop kidney disease secondary to SLE at some point in their life. Additionally, most patients with SLE develop lupus nephropathy within 5 years of diagnosis. A delay in diagnosis and treatment may predispose to Chronic Kidney Disease (CKD), increase the risk of progression to End-Stage Kidney Disease (ESKD), and increase mortality, especially in patients with lupus nephritis with a more aggressive phenotype (proliferative forms), possibly associated with other risk factors such as male sex, African-American ethnicity, genetic factors, and a high positive titer of anti-double-stranded DNA and low complement [89].

### 5.2. Pathogenesis of Lupus Nephritis

Lupus nephritis (LN) involves two key immunological processes at the glomerular level. The first is the deposition of immune complexes formed in lymphoid tissues like the lymph nodes and spleen. The second involves local formation of immune complexes when circulating autoantibodies recognize the glomerular basement membrane antigen [91]. These deposits trigger both systemic and local immune responses, with the latter being amplified and sustained by complement proteins, particularly C3. Anti-C1q antibodies, which correlate strongly with active lupus nephritis, also contribute to disease progression by binding to the glomerular membrane and activating the complement cascade [92].

The location of immune complex deposition within the glomerulus determines both the histopathological features and clinical manifestations. In the subendothelial pattern, primarily seen in ISN/RPS classes III and IV (“proliferative” forms), the deposits cause predominant endothelial injury and inflammation. This manifests clinically as hematuria, acute kidney injury (AKI), and proteinuria. In contrast, the subepithelial pattern, characteristic of ISN/RPS class V (membranous glomerulonephritis), results in predominant podocyte damage with less inflammation. These patients typically present with nephrotic-range proteinuria.

Overall, the typical presence of flares and the clear instability of the immune system in SLE makes this immunological disease a clear example of an application of the framework of immunological avalanches.

## 6. Immunological Avalanches in Kidney Transplant

**Figure 2:**
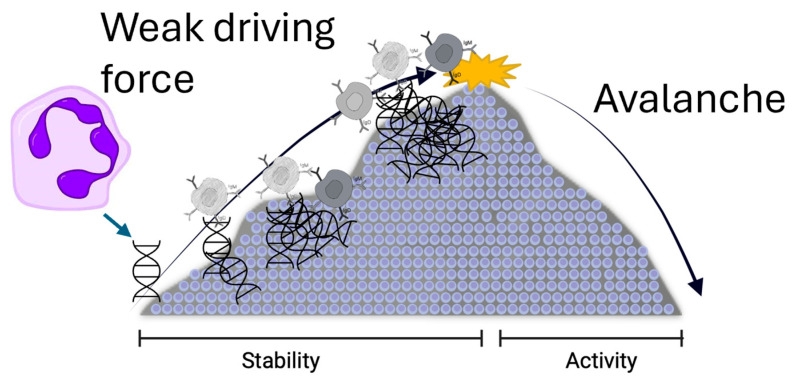
A scheme of the idea of immunological avalanche in lupus and kidney transplant. A weak, slow driving force continuously stimulates the immune system, though at a subclinical level. Here, the activation is represented as a neutrophil releasing increasing amounts of DNA, thereby activating antibody production. A feedback mechanism and the pharmacological immunomodulation keep the system at near-critical-point threshold. Under such circumstances, a seemingly small stimulus causes a large “avalanche” with rapid activation of the immune system and organ damage (lupus flare or kidney rejection).

Kidney transplantation involves a dynamic interplay between the recipient’s immune system and the transplanted organ, often characterized by oscillatory immune activity. Periods of immune quiescence can be interrupted by spontaneous activation, leading to rejection episodes. These rejection episodes can vary significantly in their presentation and impact on kidney function (see Figure 2).

Subclinical rejection occurs without noticeable symptoms and may not cause immediate changes in kidney function. Studies have reported that up to 30% of kidney transplant [93] recipients experience subclinical rejection within the first three months post-transplant. Since these episodes often go undetected without routine surveillance biopsies, relying solely on serum creatinine levels may be insufficient for early diagnosis. Research indicates that a significant proportion of subclinical rejection cases, if left untreated, can contribute to chronic allograft damage over time [93]. In contrast, clinical rejection is typically associated with overt symptoms and a marked rise in serum creatinine levels, indicating impaired kidney function. However, serum creatinine alone is not a sensitive marker for early detection, as substantial acute and chronic graft injury may already be present by the time creatinine levels significantly increase. This highlights the importance of alternative monitoring strategies, such as biomarker assessments and surveillance biopsies, to detect and manage rejection episodes before they progress to irreversible damage [94].

**Figure 3:**
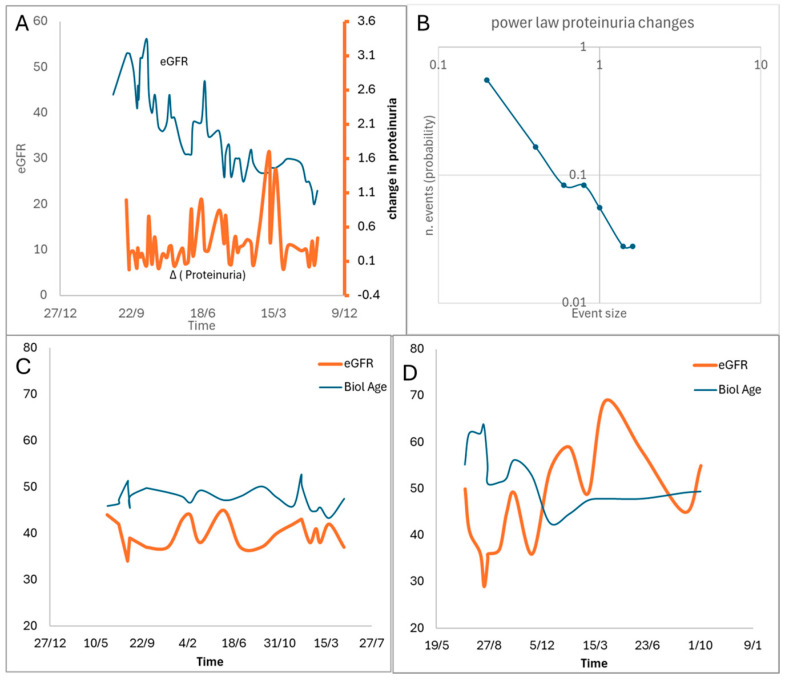
Immunological avalanches in kidney transplant. (**A**,**B**) Among the tested variables, proteinuria may be a suitable parameter to predict instability in the kidney graft (note the peaks of large proteinuria changes before the fall in eGFR). Oscillations in the proteinuria follow the power law known for near-critical-point systems (**B**). Two examples of oscillatory events in eGFR and biological age are graft stability (**C**) and progressive graft dysfunction (**D**).

To give an exemplificative picture of the transplant dynamics, in Figure 3, we report typical data from 10 transplant recipients with a mean post-transplant duration of 8 years. The monitoring period spanned 7 years with sampling intervals averaging 30 days. We tracked graft function parameters and hemochrome values while estimating epigenetic age using the Phenoage algorithm. The PhenoAge algorithm uses several biomarkers (albumin, creatinine, C-reactive protein (CRP), alkaline phosphatase, glucose, lymphocyte percentage, mean corpuscular volume, red blood cell distribution width (RDW), and white blood cell count) to estimate the epigenetic clock in a subject [95]. Sample entropy was employed to quantify the information content of temporal series. These exemplificative data show dynamic instability across all measured variables, though with differing magnitudes. The Phenoage-estimated biological age demonstrated both subtle fluctuations and marked variations that correlated with significant changes in creatinine levels. Notably, proteinuria exhibited precise log–log linearity in power law analysis, supporting the hypothesis of near-critical-point system behavior characterized by rare but significant avalanche events. It is possible to identify two distinct dynamic patterns: First, large oscillations in eGFR, proteinuria, or biological age predicted declining graft function with major avalanche events. Second, small oscillations indicated system stability. During avalanche events, biological age increased dramatically—up to 20 years above chronological age—followed by partial recovery that left a persistent 3-year elevation after each acute kidney injury episode.

This yields three significant implications for transplant monitoring: First, tracking the oscillations in creatinine and proteinuria levels provides better indication of system instability and potential avalanche events than monitoring absolute values alone. Second, biological age calculations offer valuable prognostic information. Third, proteinuria measurements serve as an efficient parameter for assessing system dynamics. These insights suggest novel approaches for monitoring transplant stability and predicting rejection episodes.

**Figure 4:**
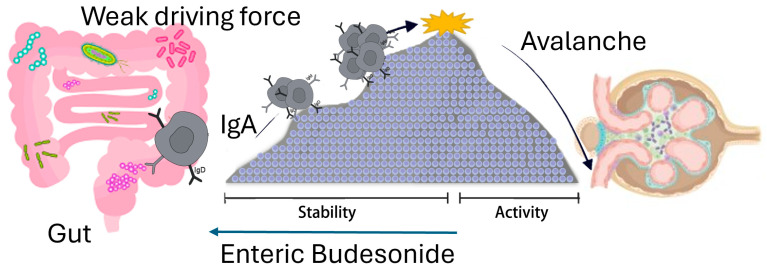
Pathogenesis of IgA immunological avalanches. A weak, continuous driving force, here depicted as a modification of the gut microbiota, stimulates clones of B cells in Peyer’s plaques (gut) to produce modified IgA antibodies. Under the continuous driver, the system operates close to a critical point after which an avalanche occurs: circulating IgA antibodies are then entrapped in the mesangium of glomeruli, inducing hematuria and proteinuria. The use of local anti-inflammatory drugs, such as enteric release budesonide, can induce the system to return to a stable state. “Stability-.activity” refer to the two phases of the immune system, depicted as a mountain. The enteric budesonide moves the system towards the stability region.

As presented in Table 1, the reader expert in nephrology will rapidly identify a third example of oscillating, avalanche-type nephrological disease, that is IgA nephropathy. In this case (see Figure 4), the weak driving force is likely located in the gut, because the use of a local steroid active exclusively at the level of the gut can reduce the instability of the immune system in IgA nephropathy [35,79]. Therefore, an alteration in the gut microbiome in a genetically susceptible individual is thought to induce activation of B cells producing circulating IgA antibodies. These will then arrive in the glomeruli, interacting with mesangial cells. Typically, this capricious disease develops in periods of rest and activation often resulting in loss of kidney function if untreated.

Understanding how biological systems respond to therapeutic protocol modifications represents a fundamental challenge in clinical medicine. One promising approach leverages the relationship between a system’s spontaneous fluctuations and its response to external perturbations—a concept rooted in fluctuation–dissipation relations.

Although initially formulated for equilibrium systems, these principles extend across diverse physical systems, including those far from critical points. Importantly, researchers have successfully adapted these concepts to nonequilibrium conditions [96,97], demonstrating their applicability to biological systems [98,99,100]. This broad utility makes fluctuation–dissipation theory a potentially powerful framework for predicting therapeutic intervention outcomes.

The underlying principle is elegantly straightforward: by analyzing natural fluctuations in specific physiological variables, we can estimate how these variables would respond to adjustments in therapeutic regimens. This approach suggests that systematic monitoring of biological fluctuations during standard care could provide clinicians with valuable predictive insights, enabling treatment optimization without requiring extensive empirical testing or potentially risky trial-and-error approaches in patient care.

## 7. Conclusions

We have presented the concept of the immunological avalanche, a progressive cascade of immune events triggered by an initial stimulus, leading to chronic inflammation and tissue damage. This concept may be applied to lupus nephritis, leading to a new perspective in the understanding of the disease. To accurately forecast proximity to clinical critical points and potential instability, extensive datasets with high-frequency temporal measurements of relevant clinical variables are essential. Currently, this level of monitoring is primarily achieved only in diabetes management, where daily blood glucose sampling is standard practice.

To extend these forecasting capabilities to immunology, we need to develop portable, cost-effective devices capable of measuring relevant biomarkers (such as creatinine, C-reactive protein, circulating DNA, or IgA) from minimally invasive finger blood samples. We believe this manuscript will generate significant interest among readers and inspire the collection of large-scale, high-quality, temporally dense datasets, which will enable the full application of avalanche mathematical modeling and critical transition theory to immunological disorders.

One reviewer highlighted an excellent opportunity for high-temporal resolution data collection: arterial blood pressure monitoring. The widespread availability of affordable automatic pressure meters would enable frequent daily measurements over extended periods (months to years), potentially through remote monitoring protocols.

Additionally, commercially available smartwatches that continuously track heart rate should be evaluated as potential early warning systems for detecting approaching instability preceding immunological avalanches. These devices offer the advantage of passive, continuous monitoring without requiring active patient participation beyond wearing the device. These readily accessible technologies may provide the temporal density of physiological data needed to identify early warning signals of critical transitions in immune function, creating an immediate pathway to implement our theoretical framework while more specialized biomarker detection devices are being developed.

## Figures and Tables

**Table 1:** Oscillating or periodic diseases. The periodicity of flares should be interpreted as the mean over a population with large variability among individuals.

Name	Periodicity of Flares	Type of System (Particles) Involved
Paroxysmal nocturnal hemoglobinuria [13]	Weeks [14]	Complement
Thrombotic microangiopathies [15] (hemolitic uremic syndrome, purpura thrombotic thrombocytopenic)	1.7 Years [16]	Complement
Sickle cell crisis	Weeks [10,17]	Erythrocytes
Alopecia areata [18]	Months [19,20]	Immune
Asthma [21]	Days to weeks [22]	Immune
Atopic dermatitis (eczema)	Days to weeks [23]	Immune
Autoimmune hepatitis [24]	Months [25]	Immune
Bulloid pemphygous	Weeks to years [26]	Immune
Chronic lymphocytic leukemia (CLL)	Years [27]	Immune
Cyclic neutropenia	21 days [28]	Immune
Eosinophilic esophagitis [29]	Months	Immune
Graves’ disease [30]	Months to years [31]	Immune
Hives (urticaria)	Hours to days [32]	Immune
IBD (Crohn’s disease and ulcerative colitis) [33]	18–32 months [34]	Immune
IgA nephropathy [35]	Months [36]	Immune
Inflammatory myopathies (e.g., dermatomyositis, polymyositis) [37]	Months [38]	Immune
Minimal change disease [39]	Months [40]	Immune
Multiple sclerosis (MS) [41]	Weeks to months [42]	Immune
Polymyalgia rheumatica (PMR)	Weeks to months	Immune
Rheumatoid arthritis (RA)	Weeks to months [7]	Immune
SLE [43]	Weeks to months	Immune
Bipolar disorder, anxiety [44]	About one year [45]	Nervous system
Chronic fatigue syndrome/myalgic encephalomyelitis [46]	Weeks to months [47]	Nervous system
Cluster headache	Weeks to months [48]	Nervous system
Epilepsy [49]	Hours to months	Nervous system
Migraine [50]	Weeks [51]	Nervous system
Parkinson’s disease [52]	Weeks to months	Nervous system
Meniere’s disease	Weeks [53]	Otoliths (?)

**Table 2:** Characteristics of example autoimmune diseases with immune avalanches.

Disease	Trigger/Initiating Factor	Immunological Dynamics	Impact
Systemic Lupus Erythematosus (SLE)	UV light, infections, and stress	Autoantibodies trigger immune complex formation, leading to complement activation and tissue inflammation.	Kidney, skin, and CNS damage; systemic flares.
Multiple Sclerosis (MS)	Infection, stress, and environmental factors [60]	T cell-mediated attack on myelin sheaths, alternating between remission and inflammatory flares.	Neurodegeneration and disability.
Asthma	Allergens and pollutants	Mast cells and eosinophils initiate acute inflammatory responses during exacerbations.	Airway constriction and inflammation.
Ulcerative Colitis (UC)	Gut microbiota imbalance [33] and dietary factors [61]	T cell activation and cytokine release create cycles of calm and severe inflammation in the colon.	Chronic inflammation, tissue damage, and increased cancer risk.
Crohn’s Disease	Gut microbiota imbalance [62] and genetic predisposition	Transmural inflammation with flares driven by dysregulated T cell responses.	Fibrosis, obstruction, and fistulas.

**Table 3:** Triggers and inhibitors of the immune system.

Triggers	Inhibitors
Environmental factors: allergens, toxins, and stress, exposure to chemicals, pollutants, or UV radiation [66]	Anti-inflammatory cytokines: IL-10 and TGF-β, IL-37 [67]
Genetic predispositions [68]	Regulatory T cells (Tregs) [69]
Viruses: EBV, CMV, and HSV [70]	B regulatory cells (Bregs) [71]
Bacteria: Mycobacterium, Streptococcus, and Helicobacter pylori [72]	Immunosuppressive drugs [73]
Fungi/parasites: Candida, Toxoplasma [74]	Vagal–immune mechanisms [75]
Pro-inflammatory microbiota (e.g., *Proteobacteria*) [76]	Endocrine homeostasis: cortisol and estrogens [77]
Dietary antigens: gluten, lactose, and undigested animal proteins [78]	Protective microbiota, e.g., *Lactobacillus* and *Faecalibacterium* [79]
Proteins exposed during cellular damage (e.g., DNA, nuclear proteins) [80]	
Persistent elevation of catecholamines [81]	
